# Understanding gender and its intersection with social stratifiers on prevention and care seeking behavior of lymphatic filariasis in Nepal

**DOI:** 10.1186/s40249-023-01126-8

**Published:** 2023-08-22

**Authors:** Abriti Arjyal, Ayuska Parajuli, Chandani Kharel, Mariam Otmani Del Barrio, Sushil Chandra Baral

**Affiliations:** 1 HERD International, Kathmandu, Nepal; 2 UNICEF, UNDP/World Bank/WHO Special Programme for Research and Training in Tropical Diseases (TDR), World Health Organization, Geneva, Switzerland

**Keywords:** Gender, Intersectionality, Lymphatic filariasis, Social stratifier, Intersectional gender analysis, Gender norm, Disease vulnerability

## Abstract

**Background:**

Lymphatic filariasis (LF) is a debilitating and painful neglected tropical disease and is one of the leading causes of permanent disability. In many countries, the intersection of gender with various social stratifiers has influenced exposure to LF and ultimately impacting the disease burden and its elimination. This study aimed to explore the influence of gender and its intersection with other social stratifiers for the prevention and care seeking behavior of LF in Nepal.

**Methods:**

This study employed qualitative research methods: in-depth interviews (IDIs) and focus group discussions (FGDs) for data collection in Bardiya, Nepal. A total of 22 IDIs (11 male, 11 female) and 2 FGDs (1 male and 1 female) were conducted with the community people between January and March 2020. The participants were purposively selected to represent different social stratifiers including age, sex, ethnicity, occupation. The data collected were analyzed using a thematic framework approach with use of intersectional gender analysis matrix.

**Results:**

The study findings revealed that men spend more time outside their household compared to women while fulfilling their roles and responsibilities, largely determined by societal expectations and gender norms. This resulted in limited access to preventive health services for men, as they often missed annual mass drug administration programme in their community and limited access to preventive methods. Further traditional occupation, specific to particular ethnicity, influenced the vulnerability to LF for certain ethnic groups. The ability to prevent exposure varied among individuals. Although women made decisions regarding the use of protective methods, it was influenced by patriarchal and gender norms. They often felt a responsibility to take care and priorities males and other family members when resources are limited. The intersectionality of gender with other social stratifiers such as marital status, ethnicity, and geographical areas influenced individual’s ability to access information related to LF and care seeking.

**Conclusions:**

Overall, the findings emphasized how access to resources, division of work, norms and values and decision-making power alone and its interaction with various social stratifiers shaped peoples’ vulnerability to disease, ability to prevent exposure and response to illness.

**Graphical Abstract:**

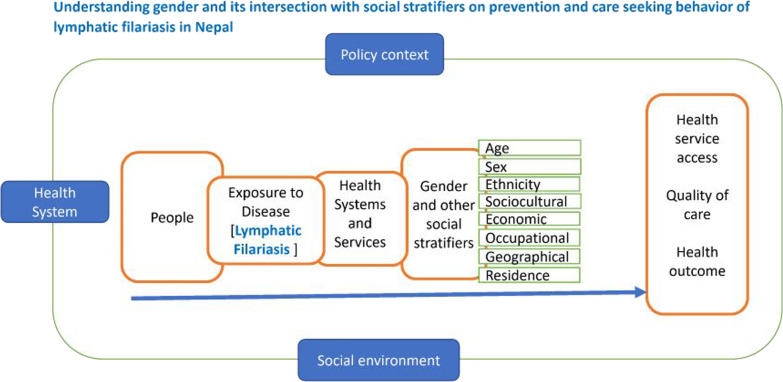

## Background

Lymphatic filariasis (LF), is a debilitating and painful neglected tropical disease (NTD) and is one of the leading causes of permanent disability. LF is caused by parasitic worms (mainly *Wuchereria bancrofti*) and transmitted by mosquitoes *Culex quinquefasciatus* as a main vector in Nepal. It is generally considered as a disease affecting poor and vulnerable populations that can result in physical impairment, loss of livelihoods or economic income leading to social stigma, exacerbated poverty and stress. Although LF has still no cure, it can be prevented through community mass drug administration (MDA) that involves annual distribution of single dose of diethylcarbamazine (DEC), in some countries replaced by ivermectin, together with albendazole irrespective of the infection status [1]. Despite achieving the Global Programme for the Elimination of Lymphatic Filariasis (GPELF) recommendation of MDA coverage for LF elimination, the high level national coverage (70.4 percent in 2018) [2] has not been proven sufficient to eliminate LF in some areas of Nepal, even after five rounds of MDA with more than 65% treatment coverage across districts. The intersection of gender with various social, cultural and political factors has profoundly influenced MDA coverage and compliance [3]. In addition, division of work within and outside the household and everyday practices influenced by sex and gender intersect with other demographics and social determinants to influence exposure to NTDs including LF, which ultimately impact on disease burden [4, 5].

According to a study, gender plays a significant role in shaping understanding of the disease transmission, coping mechanisms, and health-seeking behavior [6]. Poor understanding of causes, modes of transmission, symptoms and preventive measures of LF impairs women’s ability to protect themselves and their families from the disease [3, 7]. Stigma associated with LF is largely prevalent in the community resulting in occupational difficulty or quitting job, social exclusion, disrupted marital and social relationships [8]. The complex interaction between gender and stigma differently shapes disease experience for men and women. For example, men may feel inability to support their household, which challenges their masculinity and social role within the community [9, 10]. For women, stigma associated with NTDs can lead to increased vulnerability to gender-based violence including sexual violence, and or face different types of challenge in their marriage [9, 10], eventually, leading to psychological and mental distress and economic loss to the LF patients [11, 12].

### LF in Nepal—gender and its intersection with social stratifiers

Nepal was identified as one of the 73 LF endemic countries by World Health Organisation (WHO) where, LF has been prevalent in different topographical areas ranging from altitude of 300 feet (in the plain terai) to 5800 feet (high hill areas) above the sea level [2]. More cases are seen in Terai region when compared with hilly areas. As per WHO-Nepal, a series of LF mapping between 2001 and 2012 identified 61 of 75 districts being endemic to LF, posing risk to almost 25 million people living in these districts [13]. The baseline prevalence of LF ranged from less than 1% to 39% in these districts, with highest (39.8%) prevalence in Bardiya district [2]. The morbidity record collected during MDA from 61 districts showed that a total of 28,529 cases of LF among which majority (19,907) were hydrocele, 5704 elephantiasis and 2918 cases of hand & breast swelling and other LF manifestations [2]. A study conducted in Nepal found out higher prevalence of LF among men in comparison to women [7]. The same study identified variation in distribution of microfilarial parasite, which was highest in the age group of > 70 years and least in the ≤ 10 years. Also, morbidity pattern of this disease is directly proportional with the age of people [14].

As per commitment for global programme to eliminate LF by 2020, the Government of Nepal initiated implementation of annual MDA programme of single dose of DEC and albendazole as a preventive measure in a year 2003 and reached all 61 endemic districts by the 2013 to treat population at risk. The annual MDA campaigns for LF are conducted in all endemic districts via both house-to-house visit and fixed-post campaigns [2]. Despite good nationwide MDA coverage, LF could not be eliminated by the year 2020 from Nepal [2]. The national LF MDA coverage for the past 5 years (2013–2018) has been steady at around 70%, with 70.4% reported coverage in 2018. Certain districts, despite multiple rounds (more than 8) of MDA have not been able to eliminate LF. There exists consistent gap between coverage and compliance of MDA pills across sex, age, ethnicity, religion and educational status of the people [15]. The reason behind this is yet to be explored from an intersectional gender lens. However, accessibility and acceptability of MDA has been extensively influenced by gendered power relations and hierarchy [5, 16, 17].

In Nepal, another major challenge identified within this intervention was some educated households of urban areas preferred to receive MDA pills from health workers instead of female community health volunteers (FCHV) [18]. It is not immediately clear whether the sex of the health workers and volunteers influence this situation or whether it is a question associated with trust/mistrust in the level of education and training of FCHVs. Hence, more investigation is required on the influence of certain social stratifiers, such as sex, level of education of drug distributors and their social status on MDA acceptance among people with different social characteristics. Thus, government initiated mobilizing health workers along with FCHVs from the year 2019 in the MDA campaign.

### Intersectional gender analysis

Evidence from literature suggests that applying an intersectional gender lens is significantly important when conducting research and designing programmes on infectious diseases. Intersectional gender analysis is the process of analyzing how gender power relations intersect with other social stratifiers (such as age, ethnicity, religion, gender, education, occupation, geography, migration status, etc.) to affect people’s lives and create differences in their needs and experiences, and how policies, services and programmes can help address these differences [19]. This will help explore vulnerability to exposure of disease, health related decision making, and response to illness prevalent in the community from the perspective of gender and its interaction with other social stratifiers under given contextual factors such as discrimination, racism, classism, ableism and so on. The Government of Nepal has started to record service statistics by caste, age, ethnicity, and sex from 2014 but are not reported accordingly [20, 21]. Furthermore, other stratifiers such as education, occupation, and locality (rural/urban/remote) of the people should be wisely considered while implementing, recording, and reporting programmes to enhance the effectiveness of a health intervention including MDA programme.

Research in LF using an intersectional gender lens is limited in Nepal. Also, MDA programme data lacks disaggregated analysis on available social stratifiers, which could have masked the plight of unreached population based on their social characteristics. Further, literatures discussed above shows the influence of different social stratifiers on MDA coverage and compliance, leading to failure of eliminating LF from Nepal despite of multiple rounds of MDA in some districts [2, 4, 5, 14–18, 23]. It is acknowledged that in depth from intersectional gender perspective is needed. This would help to identify the key issues and inform the development and implementation of key strategies for LF elimination that are tailored to address gender and sociocultural related issues using intersectional perspectives in MDA programme. In order to understand the issues using intersectional gender lens a toolkit has been developed, to conduct intersectional gender analysis for infectious disease of poverty. This was a pilot study to inform the toolkit “Incorporating Intersectional Gender Analysis into Research of Infectious Diseases of Poverty” [19].

Hence, this study explores the influence of different gender domains and its intersection with other social stratifiers (i.e., age, sex, caste, ethnicity, education, religion, and occupation) for prevention and treatment (care seeking) of LF in Nepal. The gender domains that were particularly looked at include —access to resources, division of labor, roles, and everyday practices, social norms, ideologies, beliefs, and perceptions and decision-making. Further explained in Table 2.

## Methods

### Study setting

Nepal is multi-ethnic, multi-lingual, multi-religious and multi-cultural country which transitioned to a federal democratic form of government with 2015 Constitution. Health is one of the most decentralized sectors and a shared responsibility of the three tiers of government. With the progressive implementation of federalism, there has been considerable shift in terms of Nepal’s governance mechanism and structure, which is now more inclusive with decentralized decision making and authority, providing opportunity to make the health system more inclusive.

The study was conducted in Bardiya district situated in Lumbini, which lies in the western plains of Nepal and shares a border with India to its South. Administratively, Bardiya consists of six urban municipalities and two rural municipalities. There are 75 wards under these eight municipalities. As per the 2011 national census Bardiya district had a total population of approximately 0.42 million (426,576). In Bardiya, Tharus (Janajati group) are the major ethnic group followed by Chhetri/Brahmin (Pahade), Kami (Dalit), Magar (Janajati), Muslims, Yadav (Madhesi), Damai (Dalit) and Mallaha (Madhesi) [24]. Similarly, people in Bardiya predominantly follow Hindus, Muslim, Christian, Buddhist, Kirat, Bahai, etc. Bardiya is the district with highest prevalence of LF (39.8%) in Nepal. Despite nine rounds of MDA as of 2020 with at least last six rounds greater than 65% treatment coverage, Bardiya has repeatedly failed pre-transmission assessment survey to achieve LF elimination. Thus, Bardiya was selected considering the endemicity of LF and socio-cultural variation of the district, to understand the community’s perception, understanding and awareness regarding LF.

### Sampling

One ward each from two municipalities, Gulariya urban municipality (ward number 9) and Badiyataal rural municipality (ward number 3) were selected for this pilot study, to inform the toolkit to conduct intersectional gender analysis for infectious disease of poverty [19]. The municipalities and wards were selected after a consultative meeting with the district public health inspector and LF focal person considering geography, heterogeneity of the population and socio-cultural diversity. This was followed by another deliberative meeting in the selected wards with stakeholders involving local government officials, health workers from local health facility, FCHVs, schoolteachers, local political and religious leaders. Within these wards, we purposively sampled adult male and female aged 18 years and above, residing in the selected ward for over a year. The study participants were purposively sampled considering different social stratifiers such as sex, age, ethnicity, religion, marital status, education level, and occupation among others (Table 1) to conduct intersectional gender analysis for infectious disease following the WHO toolkit.

**Table 1:** Background characteristics of study participants participating in IDIs and FGDs

Characteristics	In-depth interviews (IDIs)			Focused group discussion (FGDs)	
	Male (*n* = 11)	Female (*n* = 11)	Total (*n* = 22)	Male (*n* = 8)	Female (*n* = 9)
Age, years					
≥ 18– < 20	0	0	0	1	1
20–30	5	3	8	2	3
31–40	1	2	3	2	4
41–50	1	5	6	0	1
51–60	2	1	3	2	0
61–70	2	0	2	1	0
Ethnicity					
Brahmin/Chhetri	1	1	2	1	1
Janajati	0	0	0	0	1
Dalit	2	2	4	1	1
Madesi	2	2	4	2	2
Tharu	3	3	6	3	2
Muslim	3	3	6	1	3
Religion					
Hindu	6	6	12	6	5
Muslim	3	3	6	1	3
Christian	2	2	4	1	1
Education					
Literate	11	9	20	7	9
Illiterate	0	2	2	1	0
Marital status					
Married	7	10	17	6	8
Unmarried	4	1	5	2	1
Residence					
Rural	5	6	11	8	0
Urban	4	2	6	0	3
Semiurban	2	3	5	0	6
Occupation					
Agriculture	5	3	8	2	1
Business	4	1	5	0	2
Housewife	0	4	4	0	5
Labor work	1	1	2	3	0
Student	1	1	2	2	1
Social worker	0	1	1	0	0
Pastor	0	0	0	1	0

### Data collection

The study used in-depth interviews (IDIs) and focus group discussions (FGDs) as methods for qualitative data collection. The data collection was conducted between January and March 2020. Topic guides were developed considering two frameworks: gender framework with gender power relations [25] and WHO Framework for Sex and Gender and Emerging Infectious Diseases [26]. Four major domains were used in our study: who has what (access to resources), who does what (the division of labour, roles, and everyday practices); how values are defined (social norms, ideologies, beliefs, and perceptions) and who decides (rules and decision-making). WHO Framework for Sex and Gender and Emerging Infectious Diseases [26] is based on the transmission model which explores the outbreak of a disease in relation to vulnerability to disease, exposure to pathogens, and response/treatment to illness. The framework “identifies the effects of sex and gender on the vulnerability of males and females in the population, on exposure, and on response to illness” to influence incidence, duration, and severity of disease. When using this framework, it was considered, how sex and gender intersect with other social stratifiers to influence vulnerability, exposure, and disease response. Based on these two frameworks, ‘Intersectional gender analysis matrix’, (Table 2) was used for the development of tools and analysis.

**Table 2:** Intersectional gender analysis matrix

Gender relations domains	Biological/social stratifiers						Disease domains^¥^			
	Age	Sex	Ethnicity	Marital status	Education	Occupation	Access to resources	Norms and values	Decision making power	Division of work
Vulnerability to disease	*		*	*	*	*		Women are more vulnerable to mosquito bites because of the dressing pattern		Traditional occupation increases the vulnerability as women do the household chores where men engaged in outdoor activities
Ability to prevent exposure	*		*	*	*	*	Women lacks access to information	Men and other family members prioritized for the use of bed nets	Men decide which protective measures to buy	Men miss MDA due to employment outside house
Response to illness	*	*	*	*	*	*	Women lacks access to financial resources to access health facilities		Men decide health seeking services for themselves and family members	

^¥^Illustrative examples are provided in the given framework to demonstrate how the analysis can be done. Many of the given examples can fit under more than one domain

^*^Marked are the social stratifiers that intersected with specific gender domain (column) to influence relevant infectious disease domains (row), leading to marginalisation and vulnerability of the study participant. Only those marked were found intersecting each other, and those not marked (blank) did not intersect to influence vulnerability regarding these domains

Topic guides were developed in English and translated to Nepali before data collection. Topic guides were further revised and adapted iteratively based on pretesting and field experiences. The FCHVs are active health volunteers working in their communities who helped the research team to identify the household of the participants selected during the consultative meetings. IDIs and FGDs were conducted with participants providing voluntary written consent, in a place and at a time chosen by the participants, ensuring anonymity, confidentiality and privacy. The FGDs were conducted separately for men and women in a group of 8–10. Each IDI lasted for 45 to 60 min and FGD lasted for about 90 min. Total 22 IDIs (male: 9, female: 9, male LF patients: 2, female LF patients: 2), 2 FGDs (one with male group having 8 members; another with female group having 9 members) were conducted during the study.

### Data management and analysis

Data were digitally recorded after receiving approval from the participants. The recorded data were transcribed into Nepali by the researchers, after every interview and FGD. The transcribed data were then translated to English for analysis by well-trained translators under the supervision of research team, following organizations guideline for translation. All forms of data were stored safely in HERD office in a locked cabinet and password protected computers and is accessible only to the research team members. To maintain anonymity and confidentiality, each participant was given unique code which were used for both audio recordings and the transcripts.

Thematic framework analysis was conducted with application of ‘Intersectional gender analysis matrix’, (Table 2) described in the toolkit [19] developed by WHO-TDR. A systematic iterative process was adopted to code and analyze the data from IDIs and FGDs following both deductive and inductive approach. The data was extracted into the data extraction template, which was developed according to the identified codes and themes in excel sheet in such a way that, it illustrates the data disaggregated by sex and other social stratifiers for further intersectional gender analysis. However, the template was flexible enough to adjust/create new theme and sub themes emerged during coding process, following the inductive process. Following the data coding process, themes and subthemes were further refined and explained based on the emerging data to go beyond description and look for explanatory accounts presenting similarities, discrepancies, and interlinkages within and between data. The data were analyzed looking at the linkages between ‘disease domain’ and ‘gender domain’ and the influence of social stratifiers like age, sex, ethnicity, religion, education, marital status, socio-economic status and occupation on an individual’s understanding and perception of LF (Table 2).

## Results

### Social determinants shaping vulnerability to disease

#### Occupation and its intersection with gender and other social stratifiers (ethnicity and age)

Defined gender norms and stereotypes regarding occupational roles have shaped the vulnerability to LF and ability to prevent exposure for both men and women. Gender norms dictate that men are the breadwinners of their family and women are expected to conduct the assigned household chores and responsibilities. Influenced by the gender norms and social acceptance, men and women had distinct role in livelihood activities such as agriculture and animal husbandry. Men often engaged in outdoor activities such as ploughing, decision making, selling animal products, work related internal and external migration and frequent travel to different places. Whereas women performed regular household chores such as cooking, cleaning, supporting cattle rearing, cleaning animal sheds, collecting manures, firewood, and fodder from forests. These activities made both men and women vulnerable to risk of mosquito bites and LF.

Moreover, variation in social and cultural norms for men and women, further influenced by ethnicity shaped the formation of specific behavioral factors that increased the risk of getting LF. For instance, difference in dressing habits where men work outdoors with their bodies exposed while women tend to remain covered.

“*Pattern of wearing clothes among males put them at risk as they mostly go out in shorts in summer. While females wear salwar and kurtha [tops and leggings]. We (Muslim women) always cover our face and whole body with veil*.” Female FGD, 07, Muslim.

In addition, traditional occupation linked with specific ethnicity was perceived to influence the vulnerability to LF, depending on the exposure to risk factors. For instance, people belonging to Madhesi ethnicity often reside in nearby forest areas due to their occupation involving cutting and selling wood. On the other hand, both Tharu and Madhesi communities were predominantly engaged in pig farming and agricultural activities, which increased the risk of mosquito bites and LF. As a result, most of the participant perceived individuals from Madhesi ethnic group particularly Dalit subgroups like Loath, Chamar, Teli as well as Tharu ethnicity to be more susceptible to LF than people from other ethnicities such as Pahadi.

*“I have seen LF mostly among Tharus and Desis [Madhesi] as they lack hygiene and sleep in open places. These ethnic groups are also involved in pig and duck farming as well. Hence, risk of LF is high among them.”* Male, IDI 06.

Similarly*,* considering age as a risk factor, women stated that playing in dirty areas, inability to maintain personal hygiene and dependency on parents for access to preventive measures made the children more vulnerable to LF. Likewise, elderly people being physiologically weaker, less able to take care of their personal hygiene and nutrition and neglecting their health thinking that they have spent most of their life and are close to death kept them at more risk of LF.

*“Children have high probability of getting LF because they cannot kill mosquitoes which was biting them. They play everywhere and sleep everywhere if their parents are not around them. Their parents would have to care for them when they are at home. They may get disease when they go outside the home for playing.”* Female, IDI 10.

### Influence of gender and its intersection with social stratifiers on ability to prevent exposure

#### Gender norms, access to resources and decision-making power shaping knowledge and prevention from LF

##### Use of protective measures to prevent mosquito bites

Participants were aware and used various available methods to protect themselves from mosquito bites, including the use of bed nets, mosquito coil, repellent cream and installation of wires in the doors and windows. The choice of method was influenced by factor such as need, available resources and personal and family preferences.

Bed nets were the most widely used method, considering it to be cost effective than purchasing repellants.

“*We use bed nets. We purchased bed nets for Rupees 300. However, we can’t spend Rupees 10 every day and light “mortin” (mosquito coil). When we calculate 10 rupees per day, it will be 300 rupees a month. Instead, we can use bed nets for the whole season for Rupees 300. Thus, we use it more*.” Male, FGD 02.

However, the decision-making process regarding the choice of method was sometime influenced by gender norms and perceived responsibilities. Women often felt responsible for taking care of their family members and prioritized providing bed nets to male, children, and elderly household members, when there were insufficient bed nets for everyone, despite having decision making autonomy.

*“I will give it [mosquito nets] to my husband and father-in-law [Laughs] and use the next option for me and my mother-in-law.”* Female, IDI 16.

The ethnicity and religion of participants did not influence participant’s opinion on who should be given priority for the use of bed nets.

##### MDA campaign

The government’s initiative to provide free drugs as a part of MDA campaign, to prevent LF was positively received by most of the participants. Majority of the men, women and their families had access to drugs distributed by FCHVs and health workers as part of MDA campaign. However, a few men expressed concerns about social taboos in their community related to perceived side effects of the drugs, particularly among people from Madhesi and Muslim ethnicities who still refrain from taking the drugs.

*“Community people like MDA programme because this programme is administering LF medicines to prevent LF disease at free of cost. Most of the people take medicines except few from Madhesi or Muslims due to fear of side effects.”* Male, IDI 15.

Occupational differences between men and women, also impact their access to MDA. As men spent most of the time outside the household, they face challenges in participating and miss annual MDA program and adopting preventive measures while travelling. This limits their ability to protect themselves from mosquito bites and LF.

The decision-making process regarding LF drugs intake varied among participants. Most women made decisions to participate on their own, some relied on their husband and family members.

*“My husband threatens and says, “You should take medicine. Nobody will be responsible if you get ill. One injection cost Rupees 1000. So, you must take it*.” Female, IDI 12.

#### Intersection of gender, gender roles, norms and marital status influencing access to source of information and shaping ability to prevent exposure

Participants considered LF as an incurable communicable disease caused by mosquito bites, that predominantly affects the legs resulting disfigurement resembling an elephant’s leg. Most of the men and women participants perceived lack of sanitation, poor personal hygiene and dirty living environments as risk factors for LF susceptibility. However, women LF patients were unaware about the causes and transmission of LF.

Information on LF was obtained through various sources such as television, radio, newspaper, posters and pamphlets distributed during community MDA programme, social media (such as YouTube and Facebook), school health programme and via informants. FCHVs followed by family members and friends, schoolteachers, work colleagues, health workers, neighbors, customers at shops, elected local representatives of the community and LF patients.

Access to and use of these sources for information were influenced by gender roles and norms. On the other hand, men generally believed that women have more access to media sources since they stay at home while men remain busy and mostly outside with work, had limited access.

However, gender roles associated with performing household chores and family care often limited women’s free time to engage with media sources. Women, particularly in certain ethnic groups faced restrictions within and outside their homes which limited their access to information.

*“In our community (Muslims), females are preferred only to work at house rather than going out of home. Restricted mobility to home decreases opportunity to receive information.”* Female, FGD, 09, Muslim.

The intersection of gender with marital status further complicated the women’s access to information and participation in any programs or events. After marriage women faced additional responsibilities for household chores and family care, making it more challenging to access information.

*“Unmarried people have liberty. After marriage, a female isn’t free. If there is any training and seminar, she first asks her husband whether he will allow it or not. She doesn’t participate in the programme unless her husband allows her. A [married female] is comparatively more confined to household chores. Observing married female in our community, she has to take care of household chores as well as farm work. She has to think about her husband, in-laws and other family chores. So, it is slightly difficult for married female to receive information as compared to unmarried female.*” Married Male, FGD 03.

In addition, decision making autonomy shifted with greater influence of husband and extended family members, although a certain level of autonomy in decisions making still existed depending on the situation.

*“Both of us [both husband and wife] attend such programme depending upon the time we have and busy hours. If he does not have time to attend, then he sends me. But he decides on who should go.”* Female, IDI 16, Muslim.

Differently, a few female participants expressed that married women could access more information through husbands and extended family members. They believed men had more exposure to the outside world, enabling them to access the information more easily.

#### Influence of ethnicity, education and occupation on access to information shaping ability to prevent exposure

Ethnicity was perceived to influence access to information, shaping ability to prevent exposure and vulnerability to disease among majority of the study participants. Pahadis (Brahmin/Chhetri) were considered more privileged, educated and had better access compared to Tharus, Madhesis and Muslims who were perceived less educated and more disadvantaged. These factors contributed to access to information and lack of interest in receiving information leading to lower awareness of LF and neglect of health and well-being within these communities.

“*People get to know about these things (LF and other diseases) only if they are educated. In this community, Madhesi have low education status. These days’ literacy is also higher among Tharus. But our children (Madhesi), they don’t go to school even we send them. They do not like studying. Hence, they (Madhesi) are backwards in every sector in this place. People who are educated will care and teachers may also provide information about LF disease to them and ask them to maintain cleanliness and hygiene. But those who have not gone to school will have very little information*.” Female, IDI 10, Madhesi.

In addition, men from Madhesi, Tharu and Muslim ethnicity faced difficulties in receiving information and participating in community programs due to language barriers.

“*According to my understanding, Nepali [referring to Pahadis] people may easily understand the information provided because they understand Nepali language. Whereas most of uneducated Madhesi people cannot not understand Nepali language but most of the information are given in Nepali language*.” Male, IDI 13, Muslim.

Irrespective of ethnicity age was also perceived as a factor influencing access to information and ability to prevent exposure by both male and female participants. Adults and adolescents were perceived as more educated and updated with technology while elderly individuals were less interested in receiving information on LF because they think they have lived most of their life and have less interest in taking LF medication.

*“Educated people will use mobiles but uneducated can use radio, TV if he has time. But others like newspaper, mobile cannot be used by uneducated people.”* Female, 45-year, IDI 19.

Some men believed that geographic residence was linked to ethnicity and education as Madhesi dalits, Yadavs and Muslims predominantly lived in remote areas with limited access to basic services including information whereas, Pahadis (Brahmin/Chhetri) lived in urban areas with better opportunities for services and information access.

### Social impact of LF shaped by gender and social norms and values

LF was perceived to cause physical impairment, stigma and discrimination leading to negative lived experiences for individuals with LF. This chronic condition restricted mobility, affecting an individual’s ability to work and earn their livelihood resulting in economic impacts. Additionally, it was perceived to have an impact on the mental well-being of individuals with LF.

“*We healthy person can easily go in front of others. However, a person feels uncomfortable if they have swollen limbs. They can’t easily perform their routine activities like we do. If a person remains ill for long time, they might see themselves as a burden. They would think it better to die rather than live. They won’t freely accept their freedom to live.”* Male, FGD 01.

A woman with LF shared her experience of facing challenges in performing routine activities due to pain in her swollen leg. The condition hindered her ability to do heavy work and carry out regular household chores.

A few women perceived that being infected with LF could decrease marriage opportunities for both men and women particularly unmarried women, due to the appearance and ability to perform tasks, as they face greater stigmatization within society.

“*People from her own community will say, who is going to marry her with such leg*? *Among married female, some of them might have understanding husbands, while some of them might neglect them due to their disease saying, “She looks ugly and can’t do anything now.” Even husband ignore his wife.*” Female, IDI 05.

LF patients may experience rejection of marriage proposals. A male LF patient shared,

“*Initially, I went to some other woman’s home with the marriage proposal. But, her uncle told, 'Will someone give daughter to a man like this [having LF disease]? He is a patient, and my daughter cannot take care of him. If something happens to him in future, who will look after my daughter?*” Male LF patient, IDI 25.

Further, according to the LF patients, stigma surrounding LF transmission existed within the community, where people maintain distance, although not directly expressed.

*“People maintain slight distance. They will stay in front of you but backbite later saying, “Don’t stay with her. Else, you might be infected as well.” However, in case of Amina (name changed) (Another female LF patient nearby her community), community people tell not to sit close with her.”* Female LF patient, IDI 23.

Some of the women participants mentioned avoiding contact with LF patient to prevent transmission, while others mentioned that there is no discrimination.

Some participants mentioned that female LF patients may face more social stigma and discrimination compared to male LF patients because of the perceived value of men as a breadwinner in the patriarchal society. A few participants perceived that women with LF are more stigmatized, because of the belief that they will have an unproductive life and bring bad luck to the family.

“*If a married woman has suffered from LF then they will ignore her because she is already married. And if an unmarried woman is suffering from it then they pay more attention to it because they are worried about whether they can get married or not in the future because of LF. The society tends to shun the unmarried women saying that she is a bad luck for her family wherever she goes, and she will not have a good life*”. Male, IDI 20.

Female LF patient felt being discriminated against, mainly by other women in the community, but were uncertain if it was due to their gender or other factors.

*“Who knows? The way they maintain distance and fear with me to prevent transmission, who knows whether they would do the same if any other man was infected instead of me!”* Female LF patient, IDI 22.

### Sex, gender and other social stratifiers influencing health care seeking behavior

Participants in this study had access to government and private health facilities, but some had preference for alternative practices such as Ayurvedic medicine and traditional health practices. Sex differences existed while choosing health care facilities. Most males preferred going to government health facilities as treatment was available free of cost. Few of them preferred going to local pharmacies for minor ailments. On the other hand, most of the female participants opted going to the local pharmacyor health facilities. While few, including female LF patient visited traditional healers (*witch doctors in community)* and quacks in neighboring India if the ineffective or believed that their condition was caused by supernatural powers (as being haunted by ghost/ sprit or have committed a sin).

*“There is Guruba [faith healer] who does phukphak for getting rid away from ghosts. There is also Dhami [faith healer]. Both live nearby our place. My mother goes there when God comes into her. Also, some other people from the same community go to that place. The treatment cannot be done and is not possible if a ghost is haunting. Then, we should go to Guruba, and we will be well treated. Only Guruba helps to get rid away from ghost.”* Male, IDI 15.

This was further influenced by the inter-relationship between education, ethnicity, and socio-economic status. Few participants, mentioned that educated individuals often from higher socioeconomic backgrounds, preferred private health facilities within and outside the district, while uneducated or less educated often from low socioeconomic backgrounds chose government/local health facilities or traditional healers within the community. The practice of seeking traditional health care varied based on religious beliefs. Hindus more likely preferred going to traditional healers, while Muslims and Christians preferred seeking support from religious priest or worship places for prayers.

*"Traditional healing is practiced in Hindu religion. Christians and Muslims don’t do that. Mostly elderly people, Chaudhary, Madhesi and other economically weak people visit health post. The wealthy educate their children at private school while poor people educate at government school. Same is the case with seeking health service. The wealthy go to private [clinic], while poor ones go to health post*.” Female, IDI 21.

While majority of the participants did not have specific preferences for the health workers based on their sex, one male participant mentioned that he would be more comfortable with health workers of the same sex.

*“Being a male, I prefer male doctor. Likewise, female patients prefer female doctor. In this case, male as well as female patients can express their health problems without any hesitation to the doctor.”* Male, IDI 11.

Choices of LF patients were similar and were more influenced by their economic status and social beliefs. Female LF patients did not initially seek allopathic treatment due to financial constraints, instead visited quacks and traditional healers, as it was more affordable option. On the other hand, male LF patients primarily visited doctors and occasionally visited traditional healers if allopathic medicine did not subside their symptoms.

A female LF patients had a perception that treatment by fake doctors (quacks) was more effective than visiting local health facilities.

*“When my daughter becomes sick, we bring medicine from health post sometimes. Occasionally, we also go to Gulariya (district hospital), but doctors just provide few strips of pills and write down the medicine which costs about Rupees 1000–2000. They don’t provide injection like quacks do at neighboring India, which even do not cost huge amount of money. The injection cures but pills don’t cure*.” Female LF patient, IDI 22.

She further shared her experience of visiting a traditional healer to treat LF, because of her belief system and lower cost associated with the treatment provided by the traditional healer.

*“I went for faith healing wondering if I did something wrong [against the god]. My husband is a Guru [faith healer]. People visit him around the evening time. …One of the Pahadi Guruba [male faith healer] from the locality had said, “Your leg has caught some bad spirited air. So, it has been infected. When it spreads in every 2, 4 months, the bad spirit will be moving around, your problem will increase [pain in the infected leg].”* Female LF patient, IDI 22.

Regarding decision making for seeking healthcare and purchasing medicines, most men mentioned that they made decisions for themselves and their family members. Women had varied responses, with some being the decision makers while others needed to seek permission from their husbands. Few participants mentioned taking decisions jointly, especially when it involved the health care needs of children in the family.

*“I make health related decisions mostly. I chose where to go if any family member falls ill. However, my husband earns for all of us. He supports my decision. Also, I buy medicines after checkup of sick member of family.”* Female, IDI 19.

## Discussion

In this study, most of the participants perceived LF as incurable communicable disease caused by mosquito bites primarily affecting the legs, and eventually leading to elephantiasis. The understanding of the participants from other LF endemic districts of Nepal and other countries was similar to the findings from this study [14, 27, 28]. However, participants from this study had knowledge that the certain LF symptoms such as swelling of breast, uterus and hydrocele vary between male and female due to physiological differences. This specific knowledge was not commonly identified in other studies [14, 27–30]. The increased awareness among the people residing in Bardiya may have been facilitated by multiple rounds of MDA campaigns (9 round of MDA campaigns) [2]. Further, community people and LF patients showed positive attitude and greater acceptability towards MDA campaigns, similar to a study conducted in India, which also showed increased knowledge on LF after the MDA campaigns [30]. In addition, television, radio, newspaper, posters and pamphlets distributed during community MDA programs, social media (such as YouTube and Facebook), school health programmes, FCHVs, health workers, elected local representatives and LF patients themselves were common source of common sources of information identified in this as well as others studies [14, 27, 28, 31]. However, access and use of these resources varied according to gender, age, ethnicity, marital status of women and occupation.

Gender roles and norms and its intersection with other social stratifiers influenced various aspects of disease specially in developing countries like Nepal. According to census 2011, in Nepal 43% of women and 70% of men were literate [32]. This disparity was multifaceted by factors such as gender, caste, ethnicity, geography, and disability. For example the literacy rate among women belonging to Dalit ethnic group from the Terai region is much lower at 17% [32]. Findings from this study showed that, individual’s ethnicity, linked to their geography and education influences access to information related to LF. Pahadis (Brahmin/Chhetri) mostly residing in urban areas were considered to have better access in comparison to Tharus, Madhesis and Muslims who resided in remote areas. This geographical distribution limited the access of certain ethnic groups, further exacerbating the disparities.

Nepal’s caste system categorizes certain ethnic groups as dis-advantaged based on factors such as geographical areas, access to education, disease prevalence among others [33]. In line with this categorization Madhesis were perceived to have less access to information and were less educated and backward than other ethnic groups. Moreover, linked to the previous statement this ethnic group was perceived to have poor hygiene practices at home and in their surroundings, lower usage of preventive measures to protect from mosquito bites and unhealthy lifestyles involving alcohol consumption and smoking. These factors contributed to their increased vulnerability to diseases like LF. Thus, the intertwining of various factors creates a viscous cycle of poverty related to NTDs including LF [34, 35].

Patriarchal beliefs and gendered division of labor further exacerbated the disparities in accessing information and seeking health services. Adolescent girls and women had higher school drop-out rates compared to males in Nepal due to gendered division of roles and responsibilities, where women are assigned to carry out household chores and men are associated with breadwinning, physical strength and other masculine attributes [33]. These gender roles and norms guided by patriarchal beliefs are universal to some extent, but more prevalent among lower class and less‐educated communities [18], which is similar in Nepal as well. Females face discrimination even within their families, including parent’s household due to societal norms that highly values men in a patriarchal context [33]. Additionally, median age of marriage for females of Nepal is between 17 and 19 years old [32]. Once married women have more responsibilities and more dependent on husbands and families further limiting their autonomy in accessing health care information and services [32] similar to the findings from our study. These further hindered the ability to prevent exposure and increased vulnerability to get disease for women. On the other hand, men as breadwinners of their family, are more engaged in agriculture or have to travel frequently as migrant workers. This leads to reduced access of preventive measures or LF and missed MDA campaign [5, 23, 36]. A case study from the Republic of Congo showed that occupation is linked to vulnerability to LF, with increased risk among males engaged in hunting/fishing activities and sleeping outside of the home/in the bush [37]. Similar to this our study findings identified occupation linked to ethnicity as another factor that made population from certain ethnic groups more susceptible to LF. For instance, Tharu and Madhesi communities engaged in pig farming had higher exposure to mosquito bites increasing their chances of getting LF. In alignment with other studies, [25, 26] it was perceived that different dressing habits as defined by social norms for men and women among ethnic groups, can also impact exposure as women typically wear cloths covering their bodies while and men wear short pants, increasing their vulnerability to mosquito bites.

These vulnerabilities to NTDs imposed by social and gendered norms have also been discussed in various studies. A survey conducted in Bardiya has shown a higher proportion of LF infection among males compared to females. And among the ethnic groups in the same district, the Madhesi followed by Tharu communities had the highest proportion of LF infections compared to ohter studies [25, 38, 39]. The findings from other LF endemic countries indicates that LF patients often abandon their jobs due to their disease condition leading to socioeconomic impairments [14]. This aligns with the findings of our study where restricted mobility resulting from LF was perceived as a major effect affecting individuals’ ability to work and earn livelihoods. Also LF as a chronic disease was perceived to impact on mental well-being and self-confidence as reported in other studies [14, 40]. LF patients were stigmatized because of the physical impairment caused by the diseased condition. For unmarried individuals, LF was perceived as a barrier to getting married [41]. Additionally, our study suggests irrespective of marital status; women were perceived to be more stigmatized than men likely due to societal expectations of men as bread winners. Consistent to the findings from other studies in different districts indicating a common pattern of stigmatization towards female LF Patient, in this study, community people imposed negative attitude towards female LF patients by backbiting and avoidance due to the fear of disease transmission [14]. Stigmatization can lead to social and psychological impacts on individuals leading to isolation, reduced opportunities, and diminished self-esteem.

Our study findings revealed that, female LF patients initially sought care from traditional healers and quacks and later sought care from other medical services. On the other hand, male LF patients primarily sought allopathic treatment and opted other traditional methods when allopathic measures were unsuccessful. Another study conducted in Nepal identified home-based care and ayurvedic treatment as additional health seeking behaviors among LF patients [40]. Similar to the other studies in lower income countries [4, 5]. In addition, other studies highlight the influence of gender roles and societal norms on the health seeking behavior of men and women. Women’s primary responsibility for household chores and caregiving, and lack of decision making and financial dependence on men particularly husbands limit their time and resources for seeking health care [5, 42]. Similar to other studies [16, 17], most men mentioned taking decisions for themselves and family members for seeking health care, which might have influenced women’s access to services. Irrespective of other factors, people with good socio-economic conditions visited private health facilities whereas others went to quacks and traditional healers. These variation in health facility choices align with other studies conducted in Nepal [43].

Regarding decision making for seeking healthcare and purchasing medicines, most men mentioned that they made decisions for themselves and their family members. Women had varied responses, with some being the decision makers while others needed to seek permission from their husbands. Few participants mentioned taking decisions jointly, especially when it involved the health care needs of children in the family.

This was a small pilot study conducted in one LF endemic district of Nepal. Hence, findings generated requires cautious interpretation.

## Conclusions

Findings from the study showed that people residing in Bardiya district with more than nine rounds of MDA campaigns have knowledge on LF, which included its causes, symptoms, mode of transmission, risk factors and preventive measures. Gender along with various other social stratifies namely, age, sex, ethnicity, education, occupation, socio-economic condition had major influence on perception, understanding, awareness and experience on LF of community people and LF patients. Gender domains, i.e., access to resources, division of work, norms and values and decision-making power alone and its interaction with various social stratifiers shaped peoples’ vulnerability to disease, ability to prevent exposure and response to illness. This study adds evidence on how various gender domains and social norms have shaped LF in developing country like Nepal. Evidence generated from this study illustrates that it is extremely important to break the vicious cycle of poverty due to infectious disease by incorporating gender and social determinants while designing and implementing the intervention in the respective community. Moreover, it is also important to use gendered and social lens from the aspect of disease while implementing existing programme in the community in order to identify bottlenecks of the intervention. More research is needed in other LF endemic districts of Nepal by using intersectional gender lens with the help of a toolkit titled ‘incorporating intersectional gender analysis into research of infectious diseases of poverty’. Hence, there is scope of further studies to assess the impact of gender related division of labor, everyday practices, social norms, and beliefs within and beyond the household on risk and prevalence of LF in the endemic districts.

## Data Availability

The datasets generated during this study are not publicly available due data confidentiality policy but are available from the corresponding author on reasonable request.

## Abbreviations

LF: Lymphatic filariasis
NTD: Neglected tropical disease
IDI: In-depth interview
FGD: Focus group discussion
MDA: Mass drug administration
DEC: Diethylcarbamazine
WHO: World Health Organisation
FCHV: Female community health volunteer
